# Ecological Momentary Assessment of Alcohol Marketing Exposure, Alcohol Use, and Purchases Among University Students: Prospective Cohort Study

**DOI:** 10.2196/60052

**Published:** 2024-09-03

**Authors:** Min Jin Zhang, Tzu Tsun Luk, Sai Yin Ho, Man Ping Wang, Tai Hing Lam, Yee Tak Derek Cheung

**Affiliations:** 1 School of Nursing Li Ka Shing Faculty of Medicine The University of Hong Kong Hong Kong China (Hong Kong); 2 School of Public Health Li Ka Shing Faculty of Medicine The University of Hong Kong Hong Kong China (Hong Kong)

**Keywords:** alcohol marketing, drinking, ecological momentary assessment, health behaviors, young adults, mobile phone

## Abstract

**Background:**

The relationships between alcohol marketing exposure, alcohol use, and purchase have been widely studied. However, prospective studies examining the causal relationships in real-world settings using mobile health tools are limited.

**Objective:**

We used ecological momentary assessment (EMA) to examine both the within-person– and between-person–level effects of alcohol marketing exposure on any alcohol use, amount of alcohol use, any alcohol purchase, and frequency of alcohol purchase among university students.

**Methods:**

From January to June 2020, we conducted a prospective cohort study via EMA among university students in Hong Kong who reported current drinking. Over 14 consecutive days, each participant completed 5 fixed-interval, signal-contingent EMAs daily via a smartphone app. Each EMA asked about the number and types of alcohol marketing exposures, the amount and types of alcohol used, and whether any alcohol was purchased, all within the past 3 hours. We used 2-part models, including multilevel logistic regressions and multilevel gamma regressions, to examine if the number of alcohol marketing exposure was associated with subsequent alcohol use and alcohol purchase.

**Results:**

A total of 49 students participated, with 33% (16/49) being male. The mean age was 22.6 (SD 2.6) years. They completed 2360 EMAs (completion rate: 2360/3430, 68.8%). Participants reported exposure to alcohol marketing in 5.9% (140/2360), alcohol use in 6.1% (145/2360), and alcohol purchase in 2.4% (56/2360) of all the EMAs. At the between-person level, exposure to more alcohol marketing predicted a higher likelihood of alcohol use (adjusted odd ratio [AOR]=3.51, 95% CI 1.29-9.54) and a higher likelihood of alcohol purchase (AOR=4.59, 95% CI 1.46-14.49) the following day. Exposure to more alcohol marketing did not increase the amount of alcohol use or frequency of alcohol purchases the following day in participants who used or purchased alcohol. At the within-person level, exposure to more alcohol marketing was not associated with a higher likelihood of alcohol use, amount of alcohol use, higher likelihood of alcohol purchase, or frequency of alcohol purchases the following day (all *P*_s_>.05). Each additional exposure to alcohol marketing within 1 week predicted an increase of 0.85 alcoholic drinks consumed in the following week (adjusted B=0.85, 95% CI 0.09-1.61). On days of reporting alcohol use, the 3 measures for alcohol marketing receptivity were not associated with more alcohol use or purchase (all *P*_s_>.05).

**Conclusions:**

By using EMA, we provided the first evidence for the effect of alcohol marketing exposure on initiating alcohol use and purchase in current-drinking university students. Our findings provide evidence of the regulation of alcohol marketing for the reduction of alcohol use and purchase among young adults.

## Introduction

Alcohol use is a leading risk factor for premature death and disability worldwide and has no safe level of consumption [1]. Among individuals aged 15-29 years, alcohol use is the single biggest risk factor for violence and death due to external causes and plays a major role in mental health problems such as depression and anxiety [2]. According to the social-ecological model, the associated factors of alcohol use are nested in 5 levels—individual, interpersonal, organizational, community, and policy [3]. Alcohol marketing is a major community- and policy-level factor through advertising, promotion, sponsorships, and point-of-sale display in retail [4].

The relationship between alcohol marketing and drinking behaviors has been extensively studied, particularly in Western countries. Alcohol marketing influences drinking behaviors through short-term effects [5], where repeated exposure increases familiarity and leads to more consumption and purchases, and long-term effects, where marketing normalizes alcohol use by increasing perceived social approval and emphasizing positive consequences [6]. Two systematic reviews, including 25 cross-sectional and longitudinal studies, consistently indicated a positive association between alcohol marketing exposure and intentions to drink, as well as binge or hazardous drinking [7,8]. These studies also found dose-response relationships between marketing exposure and the initiation and frequency of drinking [7,8]. However, these studies may underestimate the true impact of alcohol marketing as they often focus on advertising, neglecting other forms of promotion. Another systematic review extended the analysis to various forms of alcohol marketing, including advertising on mixed media, points of sales, and so forth, and these were positively associated with frequency and quantity of alcohol use [9]. However, using self-reported marketing exposure in the previous week or month had a large recall bias. Also, cross-sectional studies failed to establish causality. The alcohol industry argues that marketing regulations are ineffective, claiming insufficient evidence that marketing influences behavior and asserting that it only affects brand selection and market share [10].

To better understand the causal relationship between alcohol marketing and drinking behavior, several randomized controlled trials (RCTs) have been conducted [11-17]. A meta-analysis of 7 RCTs in young adults found that exposure to alcohol marketing within a 1- to 90-minute timeframe led to a small but significant increase in alcohol use (effect size: 0.20, 95% CI 0.05-0.34) [18]. However, these RCTs, which were all laboratory based with low ecological validity, often failed to account for prior exposure to alcohol marketing and involved only television marketing. This highlights the need for studies that accurately measure alcohol marketing exposure and drinking behavior in real time and explore the prospective associations between marketing exposure and drinking behavior in real-world settings.

Ecological momentary assessment (EMA) is a repeated real-time data collection method in the natural environment and allows the modeling of temporal dynamics [19]. EMA facilitates monitoring of behavioral processes in their natural context, which can minimize recall bias [20] and avoid reverse causality [21]. Our searches on PubMed and Web of Science up to July 2023, using relevant keywords of “ecological momentary assessment,” “alcohol marketing,” “alcohol advertisement,” “drinking,” “alcohol consumption,” and “alcohol use,” found only 2 studies that used EMA to assess the association between alcohol marketing exposure and normalization of alcohol use in adolescents [22,23]. One study found that more alcohol marketing exposure was associated with higher perceived social approval and popularity of alcohol use [22]. Another study found that higher adolescent perceptions of social approval and popularity of alcohol use were associated with more receptivity toward marketing exposures [23]. We found no other EMA-based studies examining the effect of alcohol marketing directly on alcohol use and purchase in adolescents and young adults.

Hong Kong has a low alcohol consumption compared to most Western countries and Asian countries such as Japan (8.0 L per capita), with a per capita consumption of 2.9 L [24,25]. A recent study found that about 30% of Hong Kong adults drank alcohol in the past 12 months [26], and Hong Kong drinkers consume 9.7 L per capita, nearly two-thirds of the per capita consumption in Japanese drinkers (14.1 L) [24]. The Hong Kong government eliminated the tax on beer and wine in 2008, which coincided with an increase in both the frequency of alcohol consumption among ever-drinkers and the prevalence of new drinkers [27,28]. This policy, combined with the high accessibility of alcohol due to no restrictions on the time and place of alcohol sales, as well as the high density of alcohol outlets, has led to an increase in alcohol consumption in the recent decade. The prevalence of past 30-day alcohol use in Hong Kong students aged 19 years and older increased from 24.2% in 2008 [29] to 33.8% in 2020 [30]. Young adults aged 18-35 years have the highest proportion of binge drinking and alcohol abuse compared to older age groups and are at high risk of various alcohol-related harms [26-28,31]. Alcohol use is also significantly associated with substance use among young adults [32,33]. A recent cross-sectional study among young adults in Hong Kong found that 71.6% were exposed to traditional marketing and 53% to social media marketing, with exposure positively associated with past-month alcohol consumption [34]. Despite this, Hong Kong imposes minimal restrictions on alcohol marketing. The only policy is a ban on alcohol advertisement on domestic free television from 4 PM to 8:30 PM [35]. Thus, Hong Kong young adults are frequently exposed to alcohol marketing in both offline and internet-based channels [36,37].

Given the extensive alcohol marketing and the surge in drinking among young adults, we aimed to examine the association between alcohol marketing and drinking behaviors to inform effective public health interventions. We used EMA to examine the effects of alcohol marketing exposure on drinking behaviors among university students. We hypothesized that first, more exposure to alcohol marketing in a day would predict any alcohol use, any alcohol purchase, increased alcohol use, and more frequent alcohol purchases the following day. This association was examined at both the within-person and between-person levels to account for intra- and interindividual variability in responses to marketing exposure, respectively. Second, more exposure to alcohol marketing in the first week would predict any alcohol use, any alcohol purchase, increased alcohol use, and more frequent alcohol purchases the following week. Finally, positive alcohol marketing receptivity would predict more alcohol use and a higher frequency of alcohol purchases the following day.

## Methods

### Study Design

This prospective study included 49 university students who participated in a 2-arm RCT (allocation ratio 1:1) to examine the discrepancy in reporting alcohol marketing exposure between EMA and conventional retrospective survey. The participants in the intervention group who completed 2 weeks of EMAs were included in this analysis.

### Procedures

We recruited participants from January to June 2020 via mass emails with a link to an internet-based anonymous survey of all undergraduate and postgraduate students in the University of Hong Kong (HKU). Potential participants interested in this project can complete the internet-based enrollment form by clicking the provided link in the mass emails. The enrollment form included questions to screen the eligibility. Students who were (1) Hong Kong residents, (2) aged 18 to 35 years, (3) enrolled in an undergraduate or postgraduate program, (4) had a smartphone with internet access, (5) drank any alcohol in the past 30 days, (6) able to read and write Chinese, and (7) stayed in Hong Kong throughout the study were eligible. A research assistant contacted the eligible participants; provided a brief overview of the study via telephone; and scheduled a face-to-face enrollment session with them in the HKU campus to obtain written consent, conduct the baseline survey, and install the EMA app on their smartphones.

### EMA Operation

Participants allocated to the intervention group were instructed to install and set up a free EMA app on their smartphones during the enrollment session. This setup included imputing a personal identification number (the last 5 digits of their phone number) and specifying the date and time they wanted to receive the first EMA. The app “HKU alcohol study” (HKU, Hong Kong Special Administrative Region of the People’s Republic of China) was developed specifically for this study. The app featured automated notifications, a customizable scheduling system, and intuitive data entry interfaces allowing participants to select options without manual text input. Alcohol marketing exposure is typically discrete. Hence, fixed-interval and signal-contingent prompts can reliably capture all exposures and behaviors, enabling comprehensive data collection on the cumulative effects of these exposures on drinking behaviors [38]. Additionally, signal-contingent EMAs are the predominant method for prompting in EMA studies, with over 77% (81/105) of EMA studies using this approach [39], as it mitigates the underreporting of alcohol events often observed in event-contingent EMAs [40]. Participants were required to complete 5 fixed-interval, signal-contingent [41] EMAs each day for 14 consecutive days, with a fixed time interval of 3 hours between each EMA. The app notified participants with a pop-up message, reminding them to use the app and complete the EMA within 5 minutes of receiving the prompt. If they did not respond, 2 additional prompts would be sent within the next 10 minutes. If they still did not respond, the corresponding EMA episode would be treated as nonresponse. The completed EMA data were immediately uploaded to the HKU server.

All participants were awarded HK $100 (US $1=HK $7.8) shopping vouchers after completing the baseline and follow-up questionnaires. Additionally, participants who completed 3 EMAs within each day would be rewarded with a HK $15 shopping voucher. An additional HK $10 shopping voucher was further provided to participants who completed all 5 EMAs within each day.

### Ethical Considerations

Ethical approval was obtained from the institutional review board of the University of Hong Kong/Hong Kong Authority Hong Kong West Cluster (UW-19-155). All participants were informed that the collected data will be kept strictly confidential and used solely for research purposes. All data were deidentified and no personal information was disclosed in this paper. Participants’ contact information was securely stored on the server located in HKU, encrypted with a password accessible only to the principal investigator and authorized research staff. Participants could be rewarded up to HK $450 for shopping vouchers in total as compensation.

### Measures

#### Alcohol Marketing Exposure

In each EMA, participants reported any exposure to alcohol marketing in the past 3 hours, except for the first episode, which enquired the exposure “since the last survey” (yes or no). Alcohol marketing includes all forms of promotional material or activities for alcoholic products; alcohol brands; or the culture of alcohol consumption such as beer festivals, wine fairs, and media of new alcoholic beverages or bars. If they reported exposure, they were asked about the number of exposures (an exposure could be counted multiple times if the same marketing was seen in various places) and the sources of exposure (including restaurants, YouTube, apps, social media, web banners, bus stations, metro stations, ads on public transport, point-of-sale at retail stores, television, newspapers, magazines, radios, and others). The number of exposures to alcohol marketing was aggregated daily, bi-daily, weekly, and 2 weeks for each participant.

#### Marketing Receptivity Toward Alcohol Marketing

For each reported exposure to alcohol marketing, participants rated their receptivity toward the marketing content using 3 items: “I like the alcohol marketing,” “I think the alcohol marketing was innovative,” and “I think the alcohol marketing was attractive” (1=strongly disagree and 5=strongly agree). For participants who reported exposure to alcohol marketing once a day, the daily marketing receptivity score for each item was the score rated by the participant for that single exposure. For participants with multiple exposures in a day, the daily score for each receptivity item was calculated by averaging the scores for all exposures on that day. These average scores were recorded as a binary variable (1 to 3: disagree and >3: agree). The “number of positive receptivity” was calculated by summing the binary variables for attractiveness, innovation, and likeability, resulting in a score from 0 to 3, indicating the number of these items the participant agreed with. Indicating “agree” to any item of marketing receptivity was treated as “any positive alcohol marketing receptivity” (yes or no) for that day.

#### Alcohol Use

In each EMA, participants reported any alcohol use in the past 3 hours, except for the first episode, which enquired about alcohol use “since the last survey” (yes or no). If they responded to alcohol use, they were asked the type of alcohol (including beer, alcopops, wine, spirits, cocktails, rice wine, Chinese spirits, Japanese sake, and others) and the number of drinks consumed. One drink is equivalent to one 330-mL bottle of beer or alcopops, one 125-mL glass of wine, one 22-mL shot of spirits or cocktails, one 180-mL glass of rice wine, or one 20-mL glass of Chinese spirits or Japanese sake. The number of drinks was aggregated daily, bi-daily, weekly, and 2 weeks for each participant. Participants who consumed at least 1 drink for the time periods (1) within the past day, (2) within the past 2 days, and (3) within the past week were classified as having had alcohol (yes or no) use for those time periods.

#### Alcohol Purchase

In each EMA episode, participants reported any purchase of alcohol in the past 3 hours, except for the first episode, which enquired about alcohol purchase “since the last survey” (yes or no). We did not ask about the quantity or frequency of alcohol purchased within each EMA episode. If they responded “yes,” it was treated as 1 instance of an alcohol purchase. The frequency of alcohol purchase was then aggregated daily, bi-daily, weekly, and 2 weeks for each participant. Participants who purchased alcohol at least once for the time periods (1) within the past day, (2) within the past 2 days, and (3) within the past week were classified as having purchased alcohol (yes or no) for those time periods.

#### Baseline Characteristics

Sex, age, study program, smoking status, age of initiating alcohol use, age of purchasing alcohol for the first time, and Alcohol Use Disorder Identification Test (AUDIT) were assessed at baseline. AUDIT is a 10-item scale (0 to 7=low-risk, 8 to 15=increasing risk, 16 to 19=harmful risk, and ≥20=probable dependence) [42,43]. AUDIT has been validated in Chinese [44]; the Cronbach α was 0.79 in this study.

### Statistical Analyses

Baseline demographic variables, smoking status, age of initiating alcohol use, age of purchasing alcohol for the first time, and AUDIT were described using percentage or mean as appropriate.

To test the first hypothesis, we used a 2-part modeling approach to address the zero-inflated and right skewed alcohol use and purchase data. First, we used multilevel logistic regressions with a random intercept to assess the prospective association between daily alcohol marketing exposure and the following day’s outcomes of (1) any alcohol use and (2) any alcohol purchase. Second, for days with reported alcohol use or purchase, multilevel gamma regression models assessed the association between daily alcohol marketing exposure and the following day’s outcomes of (1) amount of alcohol used and (2) frequency of alcohol purchase. Analyses included within-person and between-person effects, with predictor variables centered using the *xtcenter* command in Stata (StataCorp).

To test the second hypothesis, we used multivariable logistic and linear regressions to assess the effect of alcohol marketing exposure in the first week on the following week’s outcomes. Logistic regression assessed the association between alcohol marketing exposure with any alcohol use and any alcohol purchase, while linear regression assessed the association between alcohol marketing exposure with amount of alcohol use and frequency of purchases. Since participants were assessed for only 2 weeks, multilevel regression was not applied.

To test the third hypothesis, we used 2-part modeling approaches. First, multilevel logistic regressions were used to assess the association between alcohol marketing receptivity in a day (binary responses in the 3 separate items of receptivity and a total number of positive receptivity) and the following day’s outcomes. Second, for days with reported alcohol use or purchase, multilevel gamma regression models were used to assess the association between alcohol marketing receptivity in a day (binary responses in the 3 separate items of receptivity and a total number of positive receptivity) with the following day's amount of alcohol used and frequency of purchases. Sex, age, and AUDIT were adjusted for in all regressions as younger male drinkers were more likely to be exposed to alcohol marketing and drink more alcohol [45,46].

Three sensitivity analyses with the same 4 outcomes were used to supplement our result interpretation. First, in line with prior findings suggesting the effect of marketing exposure might fade out within 1.5 days [47], we performed a sensitivity analysis to evaluate the impact of a 2-day cumulative exposure on the outcomes on the subsequent day. Second, we used multivariable logistic and linear regressions to assess the cross-sectional association between alcohol marketing exposure and the 4 outcomes over 2 weeks. Third, regarding the prospective associations between alcohol marketing receptivity with alcohol use and purchase, we conducted 2 additional analyses treating the 3 separate items of alcohol marketing receptivity as continuous variables and EMAs with no marketing exposure as 0 or 3. All analyses were done using Stata (version 16.0).

## Results

### Sample Description

This study enrolled 51 participants in the EMA group. A total of 2 participants were unable to complete the 2-week EMAs due to failure to install the EMA app and receive prompts; hence, EMA data from 49 participants were analyzed. Table 1 shows that 33% (16/49) of participants were male. The mean age was 22.6 (SD 2.6) years. About 74% (36/49) had a bachelor’s degree and 18% (9/49) were current smokers. The mean age of initiating alcohol use and mean age of purchasing alcohol for the first time were 16 (SD 3.3) and 18.2 (SD 1.1) years, respectively. The mean AUDIT score was 5.8 (SD 4.5).

**Table 1:** Demographic characteristics, drinking behavior and EMA^a^ completion rate of participants (N=49).

Characteristics			Values
Age (years), mean (SD)			22.6 (2.6)
Male, n (%)			16 (33)
**Study program, n (%)**			
	Bachelor’s	36 (74)	
	Master’s or above	13 (27)	
Age of initiating alcohol use (years), mean (SD)			16.0 (3.3)
Age of purchasing alcohol for the first time (years), mean (SD)			18.2 (1.1)
Smoking in the past 30 days			9 (18)
**AUDIT^b^, mean (SD)**			5.8 (4.5)
	Low-risk drinking (1-7), n (%)	36 (74)	
	Increasing risk (8-15), n (%)	10 (20)	
	Harmful risk (16-19), n (%)	2 (4)	
	Probable dependence (≥20), n (%)	1 (2)	
**EMA completion rate, n (%)**			
	Below 50%	7 (14)	
	50%-74.9%	19 (39)	
	75% or above	23 (47)	

^a^EMA: ecological momentary assessment.

^b^AUDIT: Alcohol Use Disorders Identification Test. Total score ranged from 0 to 40, higher scores indicating higher level of alcohol dependence.

### EMA Completion Rate and Description

A total of 49 participants completed 2360 EMAs upon the 3430 prompts from the app (completion rate: 2360/3430, 68.8%). Table 2 shows that 37 (76%) of the 49 participants reported 173 exposures to alcohol marketing from various sources—restaurants (52/173, 30.1%), internet (49/173, 28.3%), public transportation (25/173, 14.5%), point-of-sale retail locations (19/173, 11%), television (14/173, 8.1%), and other places (14/173, 8.1%). On average, each participant reported 4.7 (SD 3.7) times of exposure to alcohol marketing within the 2-week EMA period. Among all alcohol marketing exposures, 38.2% (66/173) were rated as being liked, 32.9% (57/173) as innovative, and 35.2% (61/173) as attractive.

**Table 2:** Description of EMA^a^ data for alcohol marketing exposure, alcohol use, and alcohol purchase.

Variables				Values
**Total episodes of** **exposure to** **alcohol marketing, n**				140
	**Sources of alcohol marketing** **exposure (n=** **173), n (%)**			
		Restaurant	52 (30.1)	
		Internet (YouTube, app, social media, web banners, etc)	49 (28.3)	
		Transportation (bus stations, metro stations, ads on public transport, etc)	25 (14.5)	
		Point-of-sale at retail stores	19 (11.0)	
		Television	14 (8.1)	
		Others (newspapers, magazines, radios, etc)	14 (8.1)	
	**Marketing receptivity toward alcohol marketing in those exposure (n=173), n (%)**			
		I agree that I liked the alcohol marketing	66 (38.2)	
		I agree that the alcohol marketing is innovative	57 (32.9)	
		I agree that the alcohol marketing is attractive	61 (35.2)	
**Total episodes of alcohol use, n**				145
	**Type of alcohol use (n=145), n (%)**			
		Beer	51 (35.2)	
		Wine	37 (25.5)	
		Alcopops	22 (15.2)	
		Cocktails	14 (9.7)	
		Spirits	14 (9.7)	
		Japanese sake	12 (8.3)	
Total episodes of alcohol purchase, n				56

^a^EMA: ecological momentary assessment.

Table 2 also shows that 39 (80%) participants reported 145 episodes of alcohol use including beer (51/145, 35.2%), wine (37/145, 25.5%), alcopops (22/145, 15.2%), cocktails (14/145, 9.7%), spirits (14/145, 9.7%), and Japanese sake (12/145, 8.3%). On average, each participant reported 3.7 (SD 3.0) episodes of alcohol use and consumed 7.5 (SD 12.1) drinks within the 2-week EMA period. A total of 26 (53%) participants reported 56 episodes of purchasing alcohol within the 2-week EMA period.

### Association of Alcohol Marketing Exposure With Alcohol Use and Purchase

Table 3 shows that at the between-person level, exposure to more alcohol marketing predicted a higher likelihood of alcohol use (adjusted odd ratio [AOR]=3.51, 95% CI 1.29-9.54; *P*=.01) and a higher likelihood of alcohol purchase the following day (AOR=4.59, 95% CI 1.46-14.49; *P*=.01). At the within-person level, exposure to more alcohol marketing was not associated with the likelihood of alcohol use, amount of alcohol use, the likelihood of alcohol purchase, or frequency of alcohol purchase the following day (all *P*_s_>.05). The results at both the between-person and within-person levels remained robust in the 2-day cumulative exposure models.

**Table 3:** Prospective associations of alcohol marketing exposure with alcohol use and alcohol purchase on the following day (N=49).

Predictors				Outcomes						
				Any alcohol use or purchase the following day				Amount of alcohol use or purchase the following day		
				Adjusted OR^a^ per exposure (95% CI)		*P* value		Adjusted exp (B) (95% CI)		*P* value
**Alcohol use**										
	**Number of exposures to alcohol marketing within a day^b^**									
		Within-person	0.92 (0.64-1.31)^c^		.63		1.16 (0.98-1.38)^d^		.09	
		Between-person	3.51 (1.29-9.54)^c^		.01		1.89 (0.92-3.88)^d^		.08	
	**Number of exposures to alcohol marketing over the past 2 days^e^**									
		Within-person	0.86 (0.65-1.13)^f^		.27		1.13 (0.98-1.30)^g^		.07	
		Between-person	2.42 (1.45-4.03)^f^		.001		1.44 (0.97-2.13)^g^		.07	
**Alcohol purchase**										
	**Number of exposures to alcohol marketing within a day^h^**									
		Within-person	1.15 (0.75-1.76)^i^		.53		0.93 (0.85-1.02)^j^		.10	
		Between-person	4.59 (1.46-14.49)^i^		.01		1.15 (0.96-1.39)^j^		.14	
	**Number of exposures to alcohol marketing over the past 2 days^k^**									
		Within-person	1.11 (0.79-1.54)^l^		.56		1.01 (0.95-1.08)^m^		.77	
		Between-person	1.93 (0.99-3.76)^l^		.05		1.02 (0.90-1.16)^m^		.75	

^a^OR: odds ratio.

^b^The exposure is the number of alcohol marketing exposures within a day. The outcomes are any alcohol use the following day and the amount of alcohol use the following day.

^c^Multilevel logistic regression adjusted for sex, age, and baseline AUDIT (Alcohol Use Disorder Identification Test; number of observations=562).

^d^Multilevel gamma regression adjusted for sex, age, and baseline AUDIT, exclude daily alcohol use=0 (number of observations=97).

^e^The exposure is the number of alcohol marketing exposures over the past 2 days. The outcomes are any alcohol use on the subsequent day after the 2-day cumulative exposure and the amount of alcohol use on the subsequent day after the 2-day cumulative exposure.

^f^Multilevel logistic regression adjusted for sex, age, and baseline AUDIT (number of observations=532).

^g^Multilevel gamma regression adjusted for sex, age, and baseline AUDIT, exclude daily alcohol use=0 (number of observations=90).

^h^The exposure is the number of alcohol marketing exposures within a day. The outcomes are any alcohol purchase the following day and the frequency of alcohol purchases the following day.

^i^Multilevel logistic regression adjusted for sex, age, and baseline AUDIT (number of observations=562).

^j^Multilevel gamma regression adjusted for sex, age, and baseline AUDIT, exclude daily alcohol purchase=0 (number of observations=45).

^k^The exposure is the number of alcohol marketing exposures over the past 2 days. The outcomes are any alcohol purchase on the subsequent day after the 2-day cumulative exposure and the frequency of alcohol purchases on the subsequent day after the 2-day cumulative exposure.

^l^Multilevel logistic regression adjusted for sex, age, and baseline AUDIT (number of observations=532).

^m^Multilevel gamma regression adjusted for sex, age, and baseline AUDIT, exclude daily alcohol purchase=0 (number of observations=42).

Within the 2-week EMA period, 12 participants reported 0 exposure to alcohol marketing. A total of 10 participants reported no alcohol use. A total of 23 participants reported no alcohol purchase. Summing up the 3 key indicators, we identified 4 participants who did not report any exposure to alcohol marketing, alcohol use, and alcohol purchase. Therefore, we excluded these participants and ran the 2-part models of examining the prospective associations between alcohol marketing exposure with alcohol use and alcohol purchase. The results (Multimedia Appendix 1) were still consistent with Table 3.

Table 4 shows each additional exposure to alcohol marketing within 1 week predicted an increase of 0.85 alcoholic drinks consumed in the following week (adjusted B=0.85; *P*=.03). Although each additional exposure to alcohol marketing over a week was marginally associated with increased likelihood of alcohol use (AOR=1.62; *P*=.054), it was not significantly associated with likelihood of purchasing alcohol or frequency of alcohol purchases (both *P*_s_>.05, see Table 4) in the following week. Multimedia Appendix 2 shows that exposure to more marketing exposure over 2 weeks was associated with a larger amount of alcohol use (adjusted B=0.90; *P*=.02) and more frequent alcohol purchases (adjusted B=0.14; *P*=.01).

**Table 4:** Prospective associations of alcohol marketing exposure with alcohol use and alcohol purchase in the following week (N=49).

Predictors	Outcomes								
	Any alcohol use the following week^a^		Amount of alcohol use the following week^b^		Any alcohol purchase the following week^a^			Frequency of alcohol purchases the following week^b^	
	Adjusted OR^c^ per exposure (95% CI)	*P* value	Adjusted B per exposure (95% CI)	*P* value	Adjusted OR per exposure (95% CI)	*P* value	Adjusted B per exposure (95% CI)		*P* value
Number of exposures to alcohol marketing within a week	1.62 (0.99-2.65)	.054	0.85 (0.09-1.61)	.03	0.98 (0.71-1.36)	.92	0.00 (–0.18 to 0.18)		.98

^a^Multivariable logistic regression adjusted for sex, age, and baseline Alcohol Use Disorders Identification Test (number of observations=46).

^b^Multivariable linear regression adjusted for sex, age, and baseline Alcohol Use Disorders Identification Test (number of observations=46).

^c^OR: odds ratio.

### Association of Alcohol Marketing Receptivity With Alcohol Use and Purchase

Table 5 shows that on days of liking alcohol marketing, when perceiving the marketing as innovative or attractive, they were more likely to use and purchase alcohol the following day compared to days where they reported no marketing exposure, but the results were not significant (all *P*_s_>.05). On days of reporting alcohol use, the 3 measures for alcohol marketing receptivity were not associated with more alcohol use or purchase (all *P*_s_>.05). Our sensitivity analysis by treating alcohol marketing receptivity as a continuous variable showed similar results as the main analysis (Multimedia Appendix 3).

**Table 5:** Prospective association of alcohol marketing receptivity with alcohol use and alcohol purchase on the following day (N=49).

Predictors		Any alcohol use the following day^a^			Amount of alcohol use the following day^b^			Any alcohol purchase the following day^c^				Frequency of alcohol purchases the following day^d^	
		Yes, n/N (%)	Adjusted OR^e^ (95% CI)	*P* value	Adjusted exp (B) (95% CI)	*P* value	Yes, n/N (%)		Adjusted OR (95% CI)	*P* value	Adjusted exp (B) (95% CI)		*P* value
**I like the alcohol marketing**													
	No exposure	78/481 (16.2)	Ref^f^	N/A^g^	N/A	N/A	33/481 (6.9)		Ref	N/A	N/A		N/A
	No	8/59 (13.6)	0.65 (0.27-1.56)	.34	1.04 (0.58-1.88)	.89	5/59 (8.5)		1.13 (0.40-3.19)	.82	0.94 (0.77-1.14)		.52
	Yes	13/44 (29.6)	1.41 (0.63-3.15)	.40	1.03 (0.67-1.59)	.90	7/44 (15.9)		1.94 (0.73-5.17)	.19	0.89 (0.75-1.06)		.20
**The alcohol marketing was innovative**													
	No exposure	78/482 (16.2)	Ref	N/A	N/A	N/A	33/482 (6.9)		Ref	N/A	N/A		N/A
	No	9/61 (14.8)	0.67 (0.30-1.54)	.34	0.91 (0.52-1.57)	.73	6/61 (9.9)		1.29 (0.49-3.41)	.61	0.92 (0.77-1.11)		.39
	Yes	12/40 (30.0)	1.58 (0.70-3.59)	.23	1.14 (0.71-1.75)	.64	6/40 (15.0)		1.85 (0.66-5.16)	.24	0.90 (0.76-1.08)		.26
**The alcohol marketing was attractive**													
	No exposure	81/494 (16.4)	Ref	N/A	N/A	N/A	34/494 (6.9)		Ref	N/A	N/A		N/A
	No	8/47 (17.0)	0.75 (0.31-1.86)	.54	1.08 (0.60-1.92)	.81	6/47 (12.8)		1.58 (0.57-4.35)	.38	1.08 (0.60-1.92)		.81
	Yes	10/43 (23.3)	1.00 (0.42-2.28)	.99	1.12 (0.69-1.83)	.64	5/43 (11.6)		1.33 (0.45-3.90)	.61	1.12 (0.69-1.83)		.64
**Positive alcohol marketing receptivity (score range 0-3)**													
	No exposure	81/494 (16.4)	Ref	N/A	N/A	N/A	34/494 (6.9)		Ref	N/A	N/A		N/A
	0	5/35 (14.3)	0.57 (0.19-1.74)	.33	1.28 (0.61-2.67)	.52	3/35 (8.6)		1.08 (0.29-4.00)	.91	0.93 (0.72-1.20)		.60
	1	3/18 (16.7)	1.05 (0.26-4.19)	.88	0.77 (0.32-1.80)	.55	2/18 (11.1)		1.36 (0.27-6.78)	.71	0.97 (0.73-1.29)		.84
	2	2/12 (16.7)	0.64 (0.12-3.34)	.60	0.63 (0.24-1.68)	.36	2/12 (16.7)		1.80 (0.33-9.90)	.50	0.93 (0.69-1.24)		.61
	3	8/25 (32.0)	1.40 (0.50-3.87)	.59	1.31 (0.77-2.34)	.32	4/25 (16.0)		1.92 (0.56-6.56)	.30	0.87 (0.70-1.08)		.21
**Any positive alcohol marketing receptivity**													
	No exposure	81/494 (16.4)	Ref	N/A	N/A	N/A	34/494 (6.9)		Ref	N/A	N/A		N/A
	No	5/35 (14.3)	0.56 (0.18-1.70)	.31	1.23 (0.59-2.61)	.58	3/35 (8.6)		1.07 (0.29-3.99)	.92	0.94 (0.73-1.21)		.63
	Yes	13/55 (23.6)	1.10 (0.51-2.37)	.81	1.07 (0.69-1.64)	.77	8/55 (14.6)		1.7 (0.69-4.22)	.25	0.91 (0.78-1.07)		.26

^a^Multilevel logistic regression adjusted for sex, age, and baseline Alcohol Use Disorder Identification Test (AUDIT), daily alcohol use=0 versus daily alcohol use>0.

^b^Multilevel gamma regression adjusted for sex, age, and baseline AUDIT, exclude daily alcohol use=0.

^c^Multilevel logistic regression adjusted for sex, age, and baseline AUDIT, daily alcohol purchase=0 versus daily alcohol purchase>0.

^d^Multilevel gamma regression adjusted for sex, age, and baseline AUDIT, exclude daily alcohol purchase=0.

^e^OR: odds ratio.

^f^Ref: reference group.

^g^N/A: not applicable.

## Discussion

### Principal Findings

Our findings provide the first evidence that increased exposure to alcohol marketing within a single day or 2 days predicted higher likelihoods of alcohol use and purchase the following days, implying the direct effect of marketing on alcohol use and purchases. Alcohol marketing exposure in a week predicted a larger amount of alcohol use the following week, implying the accumulative effect of marketing on consumption level. Sensitivity analyses by excluding 4 participants with 0 reports of the key indicators supported the robustness of the results.

Our finding is the first to support that increased exposure to alcohol marketing was associated with a higher likelihood of alcohol use within the following day and subsequent day after 2-day cumulative exposure. This extends the evidence from previous experimental RCTs [11-17], which lacked ecological validity and only examined the immediate effects 30 minutes after the exposure. Such a direct effect is consistent with the “mere exposure effect” [5], whereby drinkers might be stimulated to initiate drinking due to greater familiarity with alcohol products due to more recent exposure to marketing. Therefore, to reduce the likelihood of alcohol use in young adults, alcohol control policies in reducing the exposure to alcohol marketing and avoiding the glamorization of drinking behavior should be implemented.

Exposure to alcohol marketing might stimulate alcohol use, but our findings showed that the exposure did not significantly increase the amount of alcohol use on the following day. We showed that 74% (36/49) of our participants have low AUDIT scores; hence, most were light drinkers and nondaily drinkers, and the marketing effect on alcohol consumption in 1 day may be small. However, we found a positive effect of weekly alcohol marketing exposure and amount of alcohol use the following week (adjusted B=0.85). Thus, the effect of accumulative alcohol marketing exposures on the amount of alcohol use assessed in the week was more detectable in this group with low alcohol consumption.

The lack of significant association between daily variations in alcohol marketing exposure and the amount of alcohol use can be attributed to the low variability in individual exposure to alcohol marketing, with only 5.9% (140/2360) of EMA episodes reporting such exposure. This limited variability in individual exposure levels results in insufficient statistical power to detect significant within-person effects. Besides, this study was conducted with the onset of the COVID-19 pandemic in Hong Kong. During this period, the government implemented numerous restrictive policies such as bar closures and dining restrictions. These restrictions likely reduced participants’ opportunities to encounter varying levels of alcohol marketing exposure, thus reducing the detectable within-person effects.

We found a positive effect of alcohol marketing exposure within a day on alcohol purchase the following day, but not on the frequency of alcohol purchase. The null association between alcohol marketing exposure in a day and the frequency of alcohol purchase the following day can be attributed to several factors. First, our EMAs did not ask for and analyze the quantity of alcohol purchased. Karaoke bars and pubs in Hong Kong often target students with discounted party packages and fixed-price “all-you-can-drink” nights, promoting larger 1-time purchases. Second, university students often consume alcohol obtained from others in social settings. A recent study has shown that students living in residence halls are twice as likely to have binge drinking compared to those living with family [48]. Third, financial constraints may limit university students’ ability to purchase alcohol frequently within a short period. Besides, our cross-sectional analysis (Multimedia Appendix 2) found that exposure to more alcohol marketing over a 2-week EMA period was associated with a higher frequency of alcohol purchases during the same period, which supported the long-term effect of alcohol marketing on purchases. Finally, reverse causation of the alcohol purchase and exposure to alcohol marketing was likely to happen.

Contrasting with previous studies that alcohol marketing receptivity might increase the frequency and amount of alcohol use [49,50], our analysis did not show sufficient evidence to support similar results at day-level. The discrepancy may be attributed to only a small number of participants who were exposed to alcohol marketing being eligible to respond to receptivity questions. In the 2360 completed EMA episodes, only 173 episodes of exposure to alcohol marketing and the corresponding marketing receptivity were assessed. It might limit the statistical power to confirm the association between marketing receptivity and alcohol use from both the main and sensitivity analyses. Further studies with a larger sample size to examine the effect of alcohol marketing receptivity and alcohol use and purchase are warranted.

Our study had a few limitations. First, the overall completion rate of EMA was about 68.8% (2360/3430), which was generally lower than previous EMA studies, which was about 76.4% on average (4 to 5 prompts per day) [51]. Future EMA studies may use a combination of event-contingent and signal-contingent prompts to capture more alcohol marketing exposure, thereby increasing the completion rate [52]. Second, the study sample was not a representative sample of all university students or young adults. Third, due to the time constraint in each EMA, we did not assess the type, quantity, and venue of alcohol purchases. Furthermore, the small sample size is unable to assess the association between different sources of alcohol marketing exposure and drinking behaviors. Our power analysis estimated the power of the between-person effect of daily marketing exposure on the likelihood of alcohol use in our study was about 22.5% (95% CI 19.9%-25.1%). Future studies with larger, more representative samples are warranted to evaluate the effects of various types of marketing exposure on drinking behavior.

### Conclusions

In conclusion, by using EMA, our study showed the direct effect of alcohol marketing exposure on initiating alcohol use and alcohol purchase in current-drinking university students in a real-world environment, refuting the claim by the alcohol industry that the marketing is only for brand promotion. Our findings provide evidence of regulating alcohol marketing for the reduction of alcohol use and purchase in young adults.

## Appendix

Multimedia Appendix 1 Sensitivity analysis for prospective associations of alcohol marketing exposure with alcohol use and alcohol purchase on the following day in participants excluding those who reported 0 incidences of alcohol marketing exposure, alcohol drinking, and alcohol purchase (n=45).

Multimedia Appendix 2 Association of alcohol marketing exposure with alcohol use and alcohol purchase over 2-week ecological momentary assessment period (N=49).

Multimedia Appendix 3 Sensitivity analysis for prospective association of alcohol marketing receptivity with alcohol use and alcohol purchase on the following day.

## Abbreviations

AOR: adjusted odd ratio
AUDIT: Alcohol Use Disorder Identification Test
EMA: ecological momentary assessment
HKU: University of Hong Kong
RCT: randomized controlled trial

## Data Availability

The data sets analyzed during this study are not publicly available due to privacy and ethical restrictions but are available from the corresponding author on reasonable request
