# Supplementary material for: Ecological Momentary Assessment of Alcohol Marketing Exposure, Alcohol Use, and Purchases Among University Students: Prospective Cohort Study
Source: JMIR Mhealth Uhealth. 2024 Sep 3;12:e60052. doi: 10.2196/60052 (PMC11408884; doi:10.2196/60052)
Supplement: Multimedia Appendix 1 [file mhealth_v12i1e60052_app1.docx]

| Predictors | Outcomes | | | | | |
| --- | --- | --- | --- | --- | --- | --- |
|  | Any alcohol use the following day^a^ | | | Amount of alcohol use the following day^b^ | | |
|  | Adjusted OR per exposure | 95% CI | *P* | Adjusted Exp(β) | 95% CI | *P* |
| Number of exposure to alcohol marketing within a day | | | | | | |
| Within-person | 0.92 | 0.64-1.31 | .64 | 1.16 | 0.98-1.38 | .09 |
| Between-person | 2.66 | 1.01-7.05 | .049 | 1.89 | 0.91-3.88 | .084 |
|  | Any alcohol use the subsequent day after the two-day cumulative exposure^c^ | | | Amount of alcohol use the subsequent day after the two-day cumulative exposure^d^ | | |
| Number of exposure to alcohol marketing over the past two days | | | | | | |
| Within-person | 0.86 | 0.65-1.13 | .269 | 1.13 | 0.99-1.30 | .071 |
| Between-person | 2.13 | 1.30-3.48 | .003 | 1.43 | 0.97-2.13 | .073 |
|  | Any alcohol purchase the following day^e^ | | | Frequency of alcohol purchases the following day^f^ | | |
| Number of exposure to alcohol marketing within a day | | | | | | |
| Within-person | 1.14 | 0.75-1.75 | .530 | 0.93 | 0.85-1.02 | .104 |
| Between-person | 3.72 | 1.20-11.52 | .023 | 1.15 | 0.96-1.35 | .136 |
|  | Any alcohol purchase the subsequent day after the two-day cumulative exposure^g^ | | | Frequency of alcohol purchases the subsequent day after the two-day cumulative exposure^h^ | | |
| Number of exposure to alcohol marketing over the past two days | | | | | | |
| Within-person | 1.10 | 0.90-3.31 | .103 | 1.01 | 0.95-1.08 | .765 |
| Between-person | 1.72 | 0.79-1.54 | .561 | 1.02 | 0.90-1.16 | .75 |

^a^Multilevel logistic regression adjusted for sex, age, baseline AUDIT (number of observations =514)

^b^Multilevel gamma regression adjusted for sex, age, baseline AUDIT, exclude daily alcohol consumption=0, (number of observations =97)

^c^Multilevel logistic regression adjusted for sex, age, baseline AUDIT, (number of observations =486)

^d^Multilevel gamma regression adjusted for sex, age, baseline AUDIT, exclude daily alcohol consumption=0, (number of observations =90)

^e^Multilevel logistic regression adjusted for sex, age, baseline AUDIT, (number of observations =514)

^f^Multilevel gamma regression adjusted for sex, age, baseline AUDIT, exclude daily alcohol consumption=0, (number of observations =45)

^g^Multilevel logistic regression adjusted for sex, age, baseline AUDIT, (number of observations =486)

^h^Multilevel gamma regression adjusted for sex, age, baseline AUDIT, exclude daily alcohol consumption=0, (number of observations =42)
