# Supplementary material for: Ecological Momentary Assessment of Alcohol Marketing Exposure, Alcohol Use, and Purchases Among University Students: Prospective Cohort Study
Source: JMIR Mhealth Uhealth. 2024 Sep 3;12:e60052. doi: 10.2196/60052 (PMC11408884; doi:10.2196/60052)
Supplement: Multimedia Appendix 2 [file mhealth_v12i1e60052_app2.docx]

| Predictors | Outcomes | | | | | |
| --- | --- | --- | --- | --- | --- | --- |
|  | Any alcohol use over 2 weeks^a^ | | | Amount of alcohol use over 2 weeks^b^ | | |
|  | Adjusted OR | 95% CI | *P* | Adjusted B | 95% CI | *P* |
| Number of exposure to alcohol marketing over 2 weeks | 1.15 | 0.86-1.56 | .33 | 0.90 | 0.14-1.66 | .02 |
|  | Any alcohol purchase over 2 weeks^a^ | | | Frequency of alcohol purchases over 2 weeks^b^ | | |
| Number of exposure to alcohol marketing over 2 weeks | 1.29 | 1.00-1.66 | .05 | 0.14 | 0.04-0.23 | .01 |

^a^Multivariable logistic regression adjusted for sex, age, AUDIT, (number of observations =47)

^b^Multivariable linear regression adjusted for sex, age, AUDIT, (number of observations =47)
