# Supplementary material for: Ecological Momentary Assessment of Alcohol Marketing Exposure, Alcohol Use, and Purchases Among University Students: Prospective Cohort Study
Source: JMIR Mhealth Uhealth. 2024 Sep 3;12:e60052. doi: 10.2196/60052 (PMC11408884; doi:10.2196/60052)
Supplement: Multimedia Appendix 3 [file mhealth_v12i1e60052_app3.docx]

Sensitivity analysis for prospective association of alcohol marketing receptivity (no marketing exposure=3) with alcohol use and alcohol purchase on the following day (N=49)

| Predictors | Any alcohol use the following day^a^ | | | Amount of alcohol use the following day^b^ | | | Any alcohol purchase the following day^a^ | | | Frequency of alcohol purchases the following day^c^ | | |
| --- | --- | --- | --- | --- | --- | --- | --- | --- | --- | --- | --- | --- |
|  | Adjusted OR | 95% CI | *P* | Adjusted exp(B) | 95% CI | *P* | Adjusted OR | 95% CI | *P* | Adjusted exp(B) | 95% CI | *P* |
| I like the alcohol marketing^d^ | | | | | | | | | | | | |
|  | 1.82 | 0.90-3.70 | .10 | 1.02 | 0.67-1.55 | .93 | 1.82 | 0.72-4.58 | .20 | 0.91 | 0.77-1.09 | .30 |
| The alcohol marketing was innovative^d^ | | | | | | | | | | | | |
|  | 1.52 | 0.84-2/75 | .17 | 1.06 | 0.73-1.55 | .75 | 1.01 | 0.45-2.27 | .97 | 0.97 | 0.83-1.13 | .70 |
| The alcohol marketing was attractive^d^ | | | | | | | | | | | | |
|  | 1.26 | 0.63-2.51 | .51 | 1.22 | 0.80-1.85 | .36 | 1.21 | 0.50-2.98 | .67 | 1.22 | 0.80-1.85 | .36 |

^a^Multilevel logistic regression adjusted for sex, age, baseline AUDIT

^b^Multilevel gamma regression adjusted for sex, age, baseline AUDIT, exclude daily alcohol consumption=0

^c^Multilevel gamma regression adjusted for sex, age, baseline AUDIT, exclude daily alcohol purchase=0

^d^Alcohol marketing receptivity was assessed on a 5-point scale, with 1 = strongly disagree, 2 = disagree, 3 = neutral, 4 = agree, 5 = strongly agree. Alcohol marketing receptivity was treated as a continuous variable, and a score of 3 was to those EMAs with no marketing exposure

Sensitivity analysis for prospective association of alcohol marketing receptivity (no marketing exposure=0) with alcohol use and alcohol purchase on the following day (N=49)

| Predictors | Any alcohol use the following day^a^ | | | Amount of alcohol use the following day^b^ | | | Any alcohol purchase the following day^a^ | | | Frequency of alcohol purchases the following day^c^ | | |
| --- | --- | --- | --- | --- | --- | --- | --- | --- | --- | --- | --- | --- |
|  | Adjusted OR | 95% CI | *P* | Adjusted exp(B) | 95% CI | *P* | Adjusted OR | 95% CI | *P* | Adjusted exp(B) | 95% CI | *P* |
| I like the alcohol marketing^d^ | | | | | | | | | | | | |
|  | 1.03 | 0.86-1.23 | .76 | 1.01 | 0.91-1.12 | .87 | 1.81 | 0.71-4.59 | .21 | 0.91 | 0.77-1.09 | .30 |
| The alcohol marketing was innovative^d^ | | | | | | | | | | | | |
|  | 1.04 | 0.86-1.25 | .71 | 1.01 | 0.91-1.13 | .81 | 0.91 | 0.39-2.11 | .83 | 0.97 | 0.83-1.13 | .70 |
| The alcohol marketing was attractive^d^ | | | | | | | | | | | | |
|  | 0.99 | 0.81-1.20 | .88 | 1.03 | 0.92-1.15 | .58 | 1.20 | 0.47-3.01 | .70 | 1.22 | .80-1.85 | .36 |

^a^Multilevel logistic regression adjusted for sex, age, baseline AUDIT

^b^Multilevel gamma regression adjusted for sex, age, baseline AUDIT, exclude daily alcohol consumption=0

^c^Multilevel gamma regression adjusted for sex, age, baseline AUDIT, exclude daily alcohol purchase=0

^d^Alcohol marketing receptivity was assessed on a 5-point scale, with 1 = strongly disagree, 2 = disagree, 3 = neutral, 4 = agree, 5 = strongly agree. Alcohol marketing receptivity was treated as a continuous variable, and coding a score of 0 to those EMAs with no marketing exposure.
